# Glial Fibrillary Acidic Protein and Ionized Calcium-Binding Adapter Molecule 1 Immunostaining Score for the Central Nervous System of Horses With Non-suppurative Encephalitis and Encephalopathies

**DOI:** 10.3389/fvets.2021.660022

**Published:** 2021-07-09

**Authors:** Gisele Silva Boos, Klaus Failing, Edson Moleta Colodel, David Driemeier, Márcio Botelho de Castro, Daniele Mariath Bassuino, José Diomedes Barbosa, Christiane Herden

**Affiliations:** ^1^Department of Veterinary Medicine, Institute of Veterinary Pathology, Justus-Liebig-Universität, Gießen, Germany; ^2^Unit of Biomathematics and Data Processing, Department of Veterinary Medicine, Justus-Liebig-Universität, Gießen, Germany; ^3^Laboratory of Veterinary Pathology, Universidade Federal do Mato Grosso, Cuiabá, Brazil; ^4^Department of Veterinary Pathology, Universidade Federal do Rio Grande do Sul, Porto Alegre, Brazil; ^5^Laboratory of Veterinary Pathology, Universidade de Brasília, Brasília, Brazil; ^6^Laboratory of Veterinary Pathology, Universidade de Cruz Alta, Cruz Alta, Brazil; ^7^Veterinary Diagnostics Center, Veterinary Institute, Universidade Federal do Pará, Castanhal, Brazil

**Keywords:** astrocytes, microglia, score system, etiology, infectious, degenerative

## Abstract

Like humans, horses are susceptible to neurotropic and neuroinvasive pathogens that are not always readily identified in histological sections. Instead, alterations in astrocytes and microglia cells can be used as pathological hallmarks of injured nervous tissue in a variety of infectious and degenerative diseases. On the other hand, equine glial cell alterations are poorly characterized in diseases. Therefore, in this study, we provide a statistically proved score system to classify astrogliosis and microgliosis in the central nervous system (CNS) of horses, based on morphological and quantitative analyses of 35 equine cases of encephalitis and/or encephalopathies and four non-altered CNS as controls. For this system, we used glial fibrillary acidic protein (GFAP) and ionized calcium-binding adapter molecule 1 (Iba1) immunohistochemistry, allied to statistical analysis to confirm that the scores were correctly designated. The scores of alterations ranged from 0 (non-altered) to 3 (severely altered) and provided a helpful method for describing astrocytic and microglial alterations in horses suffering from inflammatory and degenerative lesions. This system could be a template for comparative studies in other animal species and could aid algorithms designed for artificial intelligence methods lacking a defined morphological pattern.

## Introduction

Astrogliosis and microgliosis have been used as pathological hallmarks of injured nervous tissue in a variety of degenerative and infectious diseases (1–7). Analyses of features like quantitative and morphological alterations of astrocytes and microglia cells are commonly carried out after immunohistochemistry (IHC) employing antibodies directed against the glial fibrillary acidic protein (GFAP) and the ionized calcium-binding adapter molecule 1 (Iba1) (5, 8–11). Additionally, improvements in glial quantification techniques, also applied with these markers, through machine learning algorithms have shown a marked reduction in analysis time, increased productivity, and comparable accuracy as with manual techniques (12, 13). Astrocytic and microglial alterations have been more closely documented in neurodegenerative diseases in humans and experimental animal models, but little information is available on glial alterations in the central nervous system (CNS) of horses (14, 15). As equines are similarly susceptible to several neuroinvasive pathogens as humans (e.g., Borna disease virus, West Nile virus, and rabies virus) and might display degenerative/traumatic conditions that could mimic an infection, horses are valuable models for risk assessment of zoonoses and differential diagnosis (2, 16–20).

The establishment of a reliable quantitative and morphological classification system of astrocytic and microglial alterations in horses can help address lesion patterns on unresolved cases of encephalitis and encephalopathies. Pathologists could use the system for a complimentary assessment of lesions in the CNS and comparative studies involving glial response to injury by pathogens and/or degenerative lesions also in other animal species and humans. Meanwhile, an established score system for glial alterations would also benefit machine learning systems, as it would provide a morphological template of different stages of alterations to complement algorithms for automatic quantification.

In this light, this study aimed to establish score systems for GFAP and Iba1 immunostaining for the CNS of horses, considering the morphology and the number of astrocytes and microglia cells stained using natural cases of inflammation and degenerative lesions as a template.

## Materials and Methods

### Samples

To demonstrate the spectrum of astrocytic and microglial alterations, there were 39 CNS sections from horses available for the study. Samples were selected among necropsy cases that remained with an unclear etiologic diagnosis from horses with neurological clinical signs that do not rule out an infectious cause. These samples comprised 28 cases with inflammatory lesions suggested and/or confirmed to be caused by viruses, parasites, bacterial neurotoxins, or unspecified trigeminal ganglion inflammation; seven cases with degenerative lesions caused by trauma, bacterial neurotoxins, and possibly unknown infectious agents; and four non-altered cases used as controls (Supplementary Material 1). Complete histological assessment and etiological diagnose of each case are also available at Boos (21). Samples were retrieved from the tissue archives of Brazilian Universities related to this study's authors and from the Institute of Veterinary Pathology in Giessen (non-altered controls). Samples consisted of formalin-fixed paraffin-embedded (FFPE) material archived at room temperature and protected from light. Standardized material was unavailable for all cases due to sampling of CNS regions of interest during the necropsy of cases of natural disease and material availability after long-term storage. Therefore, CNS sections were categorized into four regions, according to Kaufmann et al. (22): (1) forebrain, (2) midbrain, (3) cerebellum, and (4) spinal cord.

### Immunohistochemistry

From each CNS region available, sections with 4-μm thickness were obtained and placed on glass slides SuperFrost® Plus Objektträger (R. Langenbrinck, Emmendingen, Germany). Slides were deparaffinized in xylol, followed by immersion in decreasing concentrations of ethanol. Endogenous peroxidase was blocked with 3% H_2_O_2_ in methanol for 20 min. To demonstrate GFAP, blocking of unspecific endogenous activity was carried out with 20% swine serum (B15-030, PAA Laboratories GmbH, Pasching, Austria) diluted in tris-buffered saline (TBS) for 10 min at room temperature. While for demonstration of Iba1, blocking was carried out with 1.5% goat serum (B11-035, PAA Laboratories GmbH, Pasching, Austria) diluted in 1% bovine serum albumin (BSA)/TBS for 1 h at room temperature. Negative controls consisting of the same CNS regions from every horse were incubated with rabbit-control serum (Dako A/S, Glostrup, Denmark) instead of primary antibody. Dilution and incubation of primary antibodies, secondary antibodies, and detection systems are described in Table 1.

**Table 1:** Immunohistochemistry for astrocytes and microglia assessment in the CNS of horses.

**Antibody**	**Source**	**Dilution**	**Secondary antibody**	**Detection system**
GFAP, polyclonal rabbit	Z0334 Dako A/S, Glostrup, Denmark	1:500 in 20% swine serum, overnight, 4°C	Swine anti-rabbit^a^, 1:100 in 20% swine serum/TBS, 30 min, RT	PAP^b^, 1:600 in 1% BSA/TBS, 30 min, RT
Iba1, polyclonal rabbit	019-19741 Wako Chemicals GmbH, Neuss, Germany	1:500 in 1% goat/serum/1% BSA/TBS, overnight, 4°C	Biot. goat anti-rabbit^c^, 1:200 in 1.5% goat serum/1% BSA/TBS, 30 min, RT	ABC^d^, 9 μl (A+B)/ml of 1.5% goat serum/1% BSA/TBS, 30 min, RT

*ABC, avidin–biotin complex; BSA, bovine serum albumin; GFAP, glial fibrillary acid protein; Iba1, ionized calcium-binding adapter molecule 1; PAP, peroxidase anti-peroxidase; RT, room temperature; TBS, tris-buffered saline*.

a *Swine anti-rabbit IgG, Z0196, Dako A/S, Glostrup, Denmark*.

b *Mouse peroxidase-anti-peroxidase (PAP) antibody, 223-005-024, Jackson ImmunoResearch Laboratories Inc., West Grove, PA, USA*.

c *Biotinylated goat anti-rabbit IgG, BA 1000, Vector Laboratories Inc., Burlingame, CA, USA*.

d *Vectastin^®^ ABC Kit peroxidase standard, PK-4000, Vector Laboratories Inc., Burlingame, CA, USA*.

#### Immunohistochemistry Quantitative Analysis

In each of the CNS regions immunostained with GFAP and with Iba1, the number of stained cells was counted in five lesioned and five non-lesioned microscopic fields magnified ×200. This would ensure that cases containing only small tissue sections would be also analyzed, while cases containing larger sections would be sufficiently represented. Microscopic fields were selected according to the histological assessment performed by Boos (21) that defined where the lesioned and non-lesioned areas were. Microscopic images were captured by a Nikon Eclipse 80i microscope (Nikon, Düsseldorf, Germany) equipped with a digital camera Nikon DS-Fi1 and analyzed with the NIS-Elements Basic Research 3.2 64bit software (Nikon, Düsseldorf, Germany).

#### Immunohistochemistry Morphologic Analysis

The morphological criteria were determined by a board-certified veterinary pathologist (CH) and a Ph.D. student (GSB). Lesioned areas were further classified in sections that predominantly displayed inflammation or degenerative lesions. Furthermore, astrocytic (5, 8) and microglial (11, 23, 24) morphologic alterations were classified from non-altered (grade 0) to severely altered (grade 3), according to Tables 2, 3. Mid-scale values were also possible. The intensity of immunostaining was not considered for the scoring system, but annotations were made when meaningful. Furthermore, as Iba1 stains, not only resident microglia but also monocytes/macrophages that migrate from the periphery, the stained cells observed within blood vessels were discarded from the scoring system. On the other hand, the pathological relevance of those cells was considered for discussion.

**Table 2:** Morphologic criteria to grade astrocytic injury in the CNS of horses according to GFAP staining.

	**Severity of astrocytic alterations**			
**Morphologic parameter**	**Non-altered**	**Mild**	**Moderate**	**Severe**
Apparent cellular proliferation	No	No or discrete	Moderate	Yes
Nucleus	No alterations	Mild increase in volume	Moderate increase in volume	Severe increase in volume, binucleated cells (gemistocytes)
Cytoplasm	Few mildly stained cells	Mildly stained	Moderately stained	Accentuated stain
Processes^a^	Long, thin, well-ramified	Long, thin, well-ramified	Long, moderately thickened	Thickened, trespassing other cells processes, glial scar formation
**Grade**	**0**	**1**	**2**	**3**

*CNS, central nervous system; GFAP, glial fibrillary acidic protein*.

a *Protoplasmic (in the gray matter, with numerous, shorter, and branched processes) and fibrous (in the white matter, with relatively few, long processes) astrocytes taken into consideration according to Sofroniew and Vinters (5)*.

**Table 3:** Morphologic criteria to grade microglial proliferation/invasion in the CNS of horses according to Iba1 staining.

	**Severity of microgliosis**			
**Morphologic parameter**	**Non-altered**	**Mild**	**Moderate**	**Severe**
Apparent cellular proliferation	No	No or discrete	Moderate	Yes
Nucleus	No alterations	Mild increase in volume	Moderate increase in volume	Severe increase in volume
Cytoplasm	Few mildly stained cells	Mildly stained	Moderately stained	Accentuated stain or vacuolated (Gitter cell)
Processes	Long, thin, well-ramified	Long, thin, well-ramified	Long, moderately thickened (bushy cells)	Thickened and/or shortened, amoeboid shape, bipolar (rod cells)
**Grade**	**0**	**1**	**2**	**3**

*CNS, central nervous system; Iba1, ionized calcium-binding adapter molecule 1*.

### Statistical Analysis

Statistical tests were conducted with the statistical program packages BMDP/Dynamic Release 8.1 (Statistical Solutions Ltd., Cork, Ireland) (25) and StatXact 9.0 (26). According to the IHC quantitative and morphologic analysis, data were sorted with each of the four CNS regions available. Mean values, standard deviation, minima, maxima, and sample sizes were calculated and tabulated from quantitative analysis. In the semiquantitative variables, the data description was carried out by specifying the medians, the quartiles (Q1 and Q3), and the smallest and largest observations with the presentation in box and whisker plot. Graphic illustrations were generated with MS Excel.

#### Statistical Analysis of the Number of Glial Fibrillary Acidic Protein+ and Ionized Calcium-Binding Adapter Molecule 1+ Cells in Different Lesion Setups

Since the data included two repeated measurements per horse (lesioned and non-lesioned area), a *t*-test for dependent samples was conducted to determine if the mean number of GFAP+ and Iba1+ cells occurred differently between lesioned and non-lesioned areas of each CNS region. In the next analysis step, only the lesioned areas were kept in the data set. To determine if the mean number of GFAP+ and Iba1+ cells was significantly different with a different morphologic alteration grade (inflammatory infiltration and degenerative lesions), the Wilcoxon–Mann–Whitney test (WMWT) and the WMWT with exact inference were applied. Statistical significance was assigned as *p* ≤ 0.05.

#### Statistical Analysis of Astrocytic and Microglial Grades of Alteration in Different Lesion Setups

Due to the ordinal scale as well as the high number of tied ranks in the data and the fact that data included two repeated measurements per horse (lesioned and non-lesioned area), the exact Wilcoxon signed-rank test was carried out. It was used to determine if there are differences in the astrocytic/microglial grade of lesioned and non-lesioned areas. To characterize the association between the range of astrocytes/microglial cells stained and the ordinally scaled astrogliosis/microgliosis grade, Spearman's rank correlation coefficient was computed. Also, statistical significance was assigned as *p* ≤ 0.05.

## Results

### Glial Fibrillary Acidic Protein Immunohistochemistry

#### Morphologic Analysis

**Figure 1:**
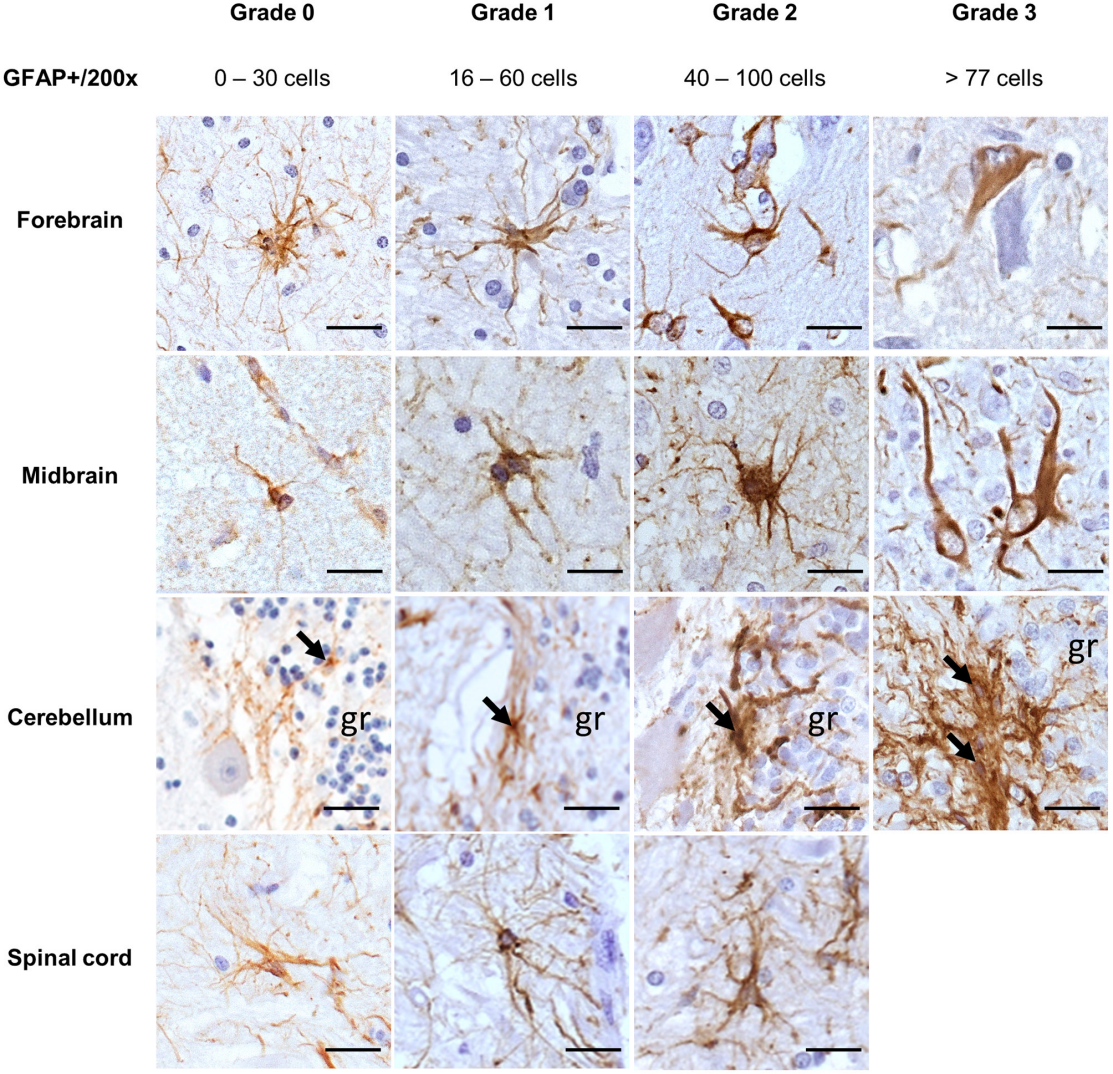
Immunohistochemical demonstration of GFAP in the CNS of horses. Grades of astrocytic alterations were established through quantitative and morphological analysis of GFAP+ cells. DAB, ×200 magnification. Spinal cord sections available for the study had no severe alterations (grade 3). gr, granular layer; GFAP, glial fibrillary acidic protein; CNS, central nervous system. Scale bar: 50 μm.

Morphologic alterations in astrocytes were compared with the immunostaining observed in the respective brain areas from four horses' non-altered CNS. Normal astrocytes from these controls were characterized by a light brown cytoplasmic GFAP staining, surrounded by long and thin processes, while the nucleus remained unstained (Figure 1, grade 0). Mild astrocytic alterations were characterized by cells with a mildly enlarged nucleus, with cytoplasm and processes still mildly stained (Figure 1, grade 1). As opposed to that, in brain regions presenting moderate (Figure 1, grade 2) and severe (Figure 1, grade 3) astrocytic alterations, there were marked morphologic alterations, such as nuclear enlargement and thickening of processes, allied to stronger immunostaining and gemistocytes observed in grade 3 (horses 4 and 29). In some cases of grade 3, the cellular nucleus appeared vacuolated (Figure 1, midbrain). However, in the spinal cord, none of the samples studied displayed morphological alterations compatible with grade 3. The range of GFAP+ cells found in each grade of alteration is demonstrated in Figure 1.

Regarding a possible pathological relation, for cases with inflammation (28/35), 14/28 had a viral infection, which displayed mostly mild-to-moderate astrocytic alterations (10/14), followed by severe alterations in 4/14. There were moderate alterations in protoplasmic and fibrous astrocytes of all cases of parasitic infections, while cases of local ganglion inflammation had mild astrocytic alterations in the CNS. Cases suggestive of bacterial neurotoxin lesions were represented by inflammatory and degenerative lesions, presenting all alteration grades. Traumatic degenerative lesions and unknown infectious pathogens causing degeneration presented moderate alterations (Figure 1).

#### Determination of the Number of Glial Fibrillary Acidic Protein+ Cells in Different Lesion Setups

**Figure 2:**
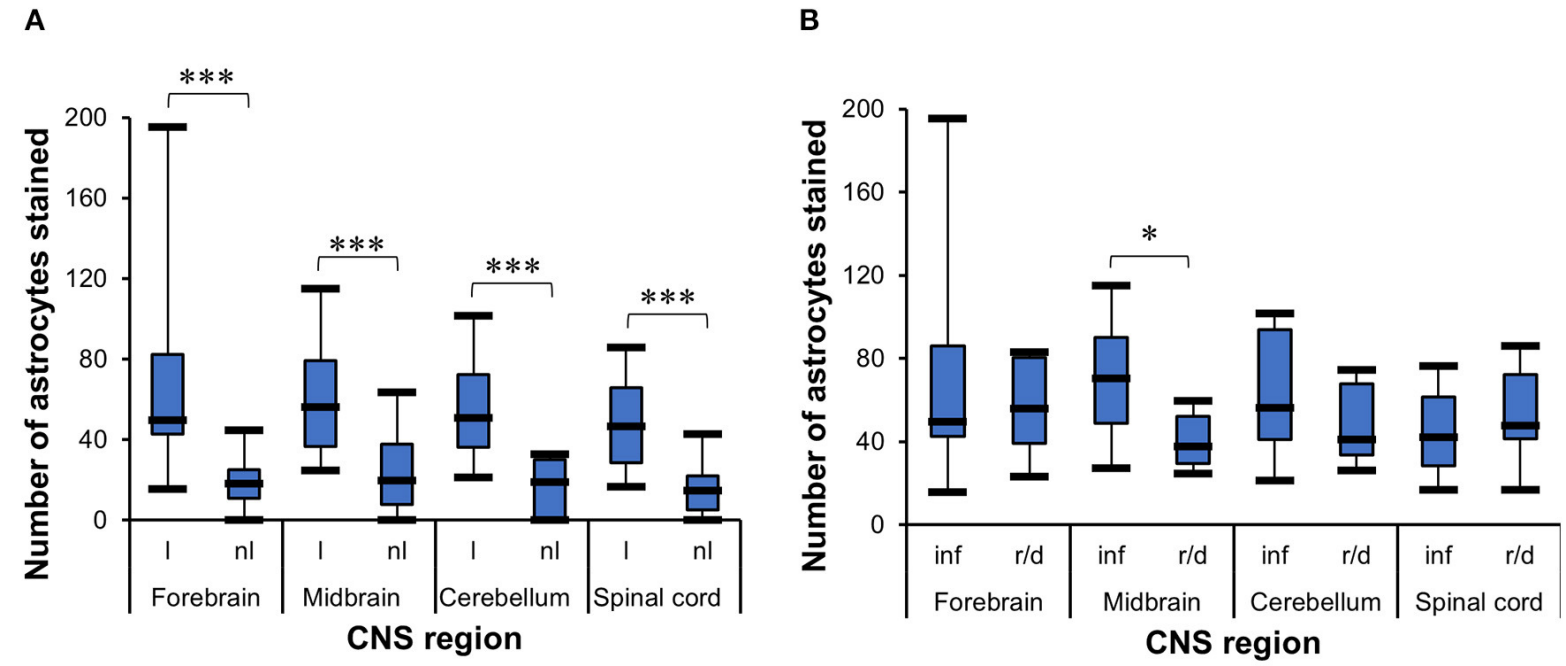
Statistical analysis of GFAP+ cells in the CNS of horses. **(A)** *t*-Test for dependent samples showed a statistically significant increase in the mean of stained astrocytes in lesioned areas compared with non-lesioned areas from all CNS regions investigated. The number of stained cells was counted in five lesioned and five non-lesioned microscopic fields magnified ×200. ***p ≤ 0.001. l, lesioned area; nl, non-lesioned area. **(B)** Wilcoxon–Mann–Whitney test demonstrated the statistically significant difference between the median number of stained astrocytes in inflammatory lesions and reactive/degenerative lesions in five microscopic fields magnified at ×200 in the midbrain. *p ≤ 0.05. inf, inflammatory lesion; r/d, reactive and/or degenerative lesion; GFAP, glial fibrillary acidic protein; CNS, central nervous system.

The *t*-test for dependent samples showed a statistically significantly higher number of GFAP+ cells in lesioned areas than in non-lesioned areas from all four CNS regions of all 35 horses investigated, namely, the forebrain [*t*_(24)_ = 6.3, *p* < 0.001], midbrain [*t*_(13)_ = 4.22, *p* = 0.001], cerebellum [*t*_(11)_ = 5.26, *p* < 0.001], and spinal cord [*t*_(21)_ = 11.01, *p* < 0.001] (Figure 2A). The median number of GFAP+ cells among the 35 horses was also higher in inflammatory lesions (median = 70.4) than in degenerative lesions (median = 37.8) in the midbrain (*U* = 38.5, *p* < 0.05), demonstrated with the WMWT (Figure 2B).

#### Comparison of Astrocytic Grades of Alteration in Different Lesion Setups

**Figure 3:**
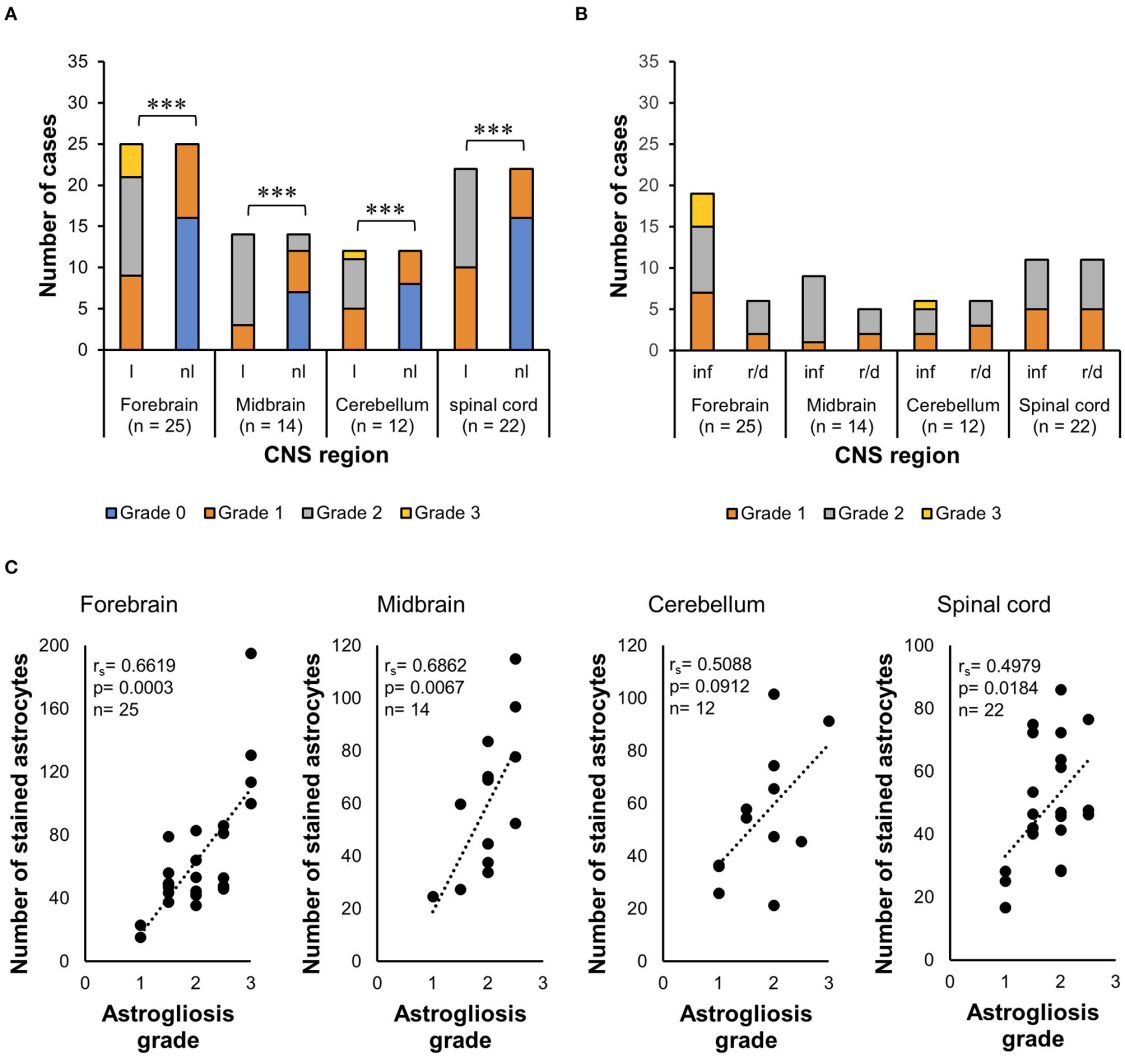
Analysis of GFAP immunohistochemistry for astrocytosis grade in the CNS of horses. **(A)** Wilcoxon signed-rank test demonstrated an increase in the grade of morphologic alterations in lesioned areas from all CNS regions, compared with the non-lesioned areas. ***p ≤ 0.001. l, lesioned area; nl, non-lesioned area; n, number of sections available from each region. **(B)** Wilcoxon–Mann–Whitney test with exact inference shows no statistically significant difference between the astrogliosis grades observed in inflammatory lesions from reactive/degenerative lesions. inf, inflammatory lesions; r/d, reactive and/or degenerative lesions. **(C)** Spearman's rank correlation coefficient shows the positive relationship between the increase in the mean of stained astrocytes and the increase in astrocytosis grade. r_s_, Spearman's correlation coefficient; GFAP, glial fibrillary acidic protein; CNS, central nervous system.

The Wilcoxon signed-rank test indicated that lesioned areas presented statistically significantly higher grades of morphologic alteration than non-lesioned areas in all CNS regions (*p* < 0.001) in general in all 35 cases (Figure 3A). The WMWT with exact inference demonstrated that the grade of astroglial activation was not statistically significantly different between inflammatory and degenerative lesions (*p* ≥ 0.05) (Figure 3B). Spearman's rank correlation coefficient revealed that there was, in general, a positive correlation between the increase in the mean of GFAP+ cells and the increase in grade of astrocytic activation in lesioned brain areas (Figure 3C). Stronger correlations were observed in the forebrain and midbrain regions. Mid-scale grades like 1.5 and 2.5 were also observed and could indicate the continuous morphological changes in the tissue, with different stages of alteration in the same area.

### Ionized Calcium-Binding Adapter Molecule 1 Immunohistochemistry

#### Morphologic Analysis

**Figure 4:**
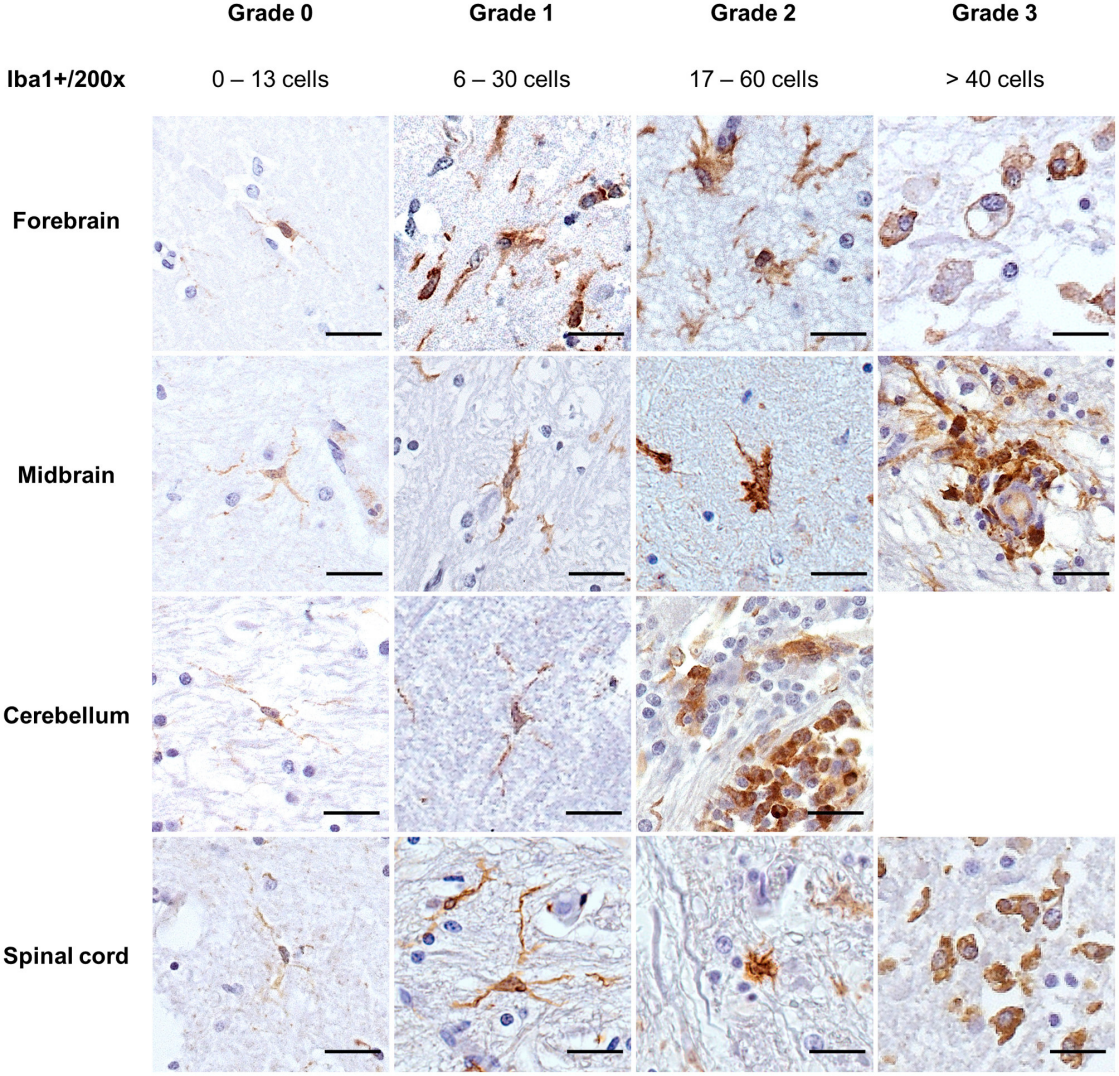
Immunohistochemical demonstration of Iba1 in the CNS of horses. Morphologic grades of microglial activation were established through quantitative and morphological analyses of Iba1+ cells. DAB, ×200 magnification. Sections of the cerebellum available for the study did not display severe microglial alterations (grade 3). Scale bar: 50 μm. Iba1, ionized calcium-binding adapter molecule 1.

Iba1 demonstrated resting microglia in the CNS in the four non-altered controls and 6/35 cases (Figure 4, grade 0). Microglia cells were scattered and light-stained, with ramified cytoplasm and oval to elongated or bean-shaped nuclei. Mild microglial alterations were associated with a slight thickening of cellular processes and nucleus rounding. In these cases, a mildly darker cytoplasm was also observed (Figure 4, grade 1). For moderate alterations, microglia cells had “bushy” features, but most of the cells still presented ramifications (Figure 4, grade 2). On the other hand, severely altered Iba1+ cells had retracted ramifications, and the cells had an amoeboid shape and were deeply stained (Figure 4, grade 3). Morphology compatible with Gitter cells was demonstrated by cells with bulky cytoplasm strongly stained on the borders, decreasing in intensity when reaching the cell center, while the nucleus, when visible, was unstained (Figure 4, grade 3, forebrain). In general, in grade 3 of alteration, it was impossible to differentiate microglia from macrophages recruited from the periphery when they were amidst the tissue; in some cases, groups of densely packed Iba1+ cells were seen within vessels, also in grade 3 tissues.

Regarding the pathology associated with the cases studied, most of the viral infections presented at least one region of the CNS with mild microglial activation (12/14 cases). This mild activation was accompanied by resting microglia (6/12) or by grade 2 microglia (2/12). There was one case with moderate microglial activation (horse 29) and another one with severe alterations (horse 27) (Supplementary Material 1). For parasitic infections, there were two distinctive stages: in 3/6 cases that were characterized with mild inflammatory lesions, there was also mild microgliosis, while in the other 3/6 cases with severe inflammatory lesions, microgliosis ranged from moderate to severe. Cases of local ganglion inflammation presented only resting microglia in the CNS. Similarly, 3/6 cases suggested to have suffered from bacterial neurotoxins showed non-altered microglia, while the other 3/6 had grade 1 alterations. Traumatic degenerative lesions and unknown infectious pathogens causing degeneration presented moderate-to-severe and mild microglial activation, respectively.

**Figure 5:**
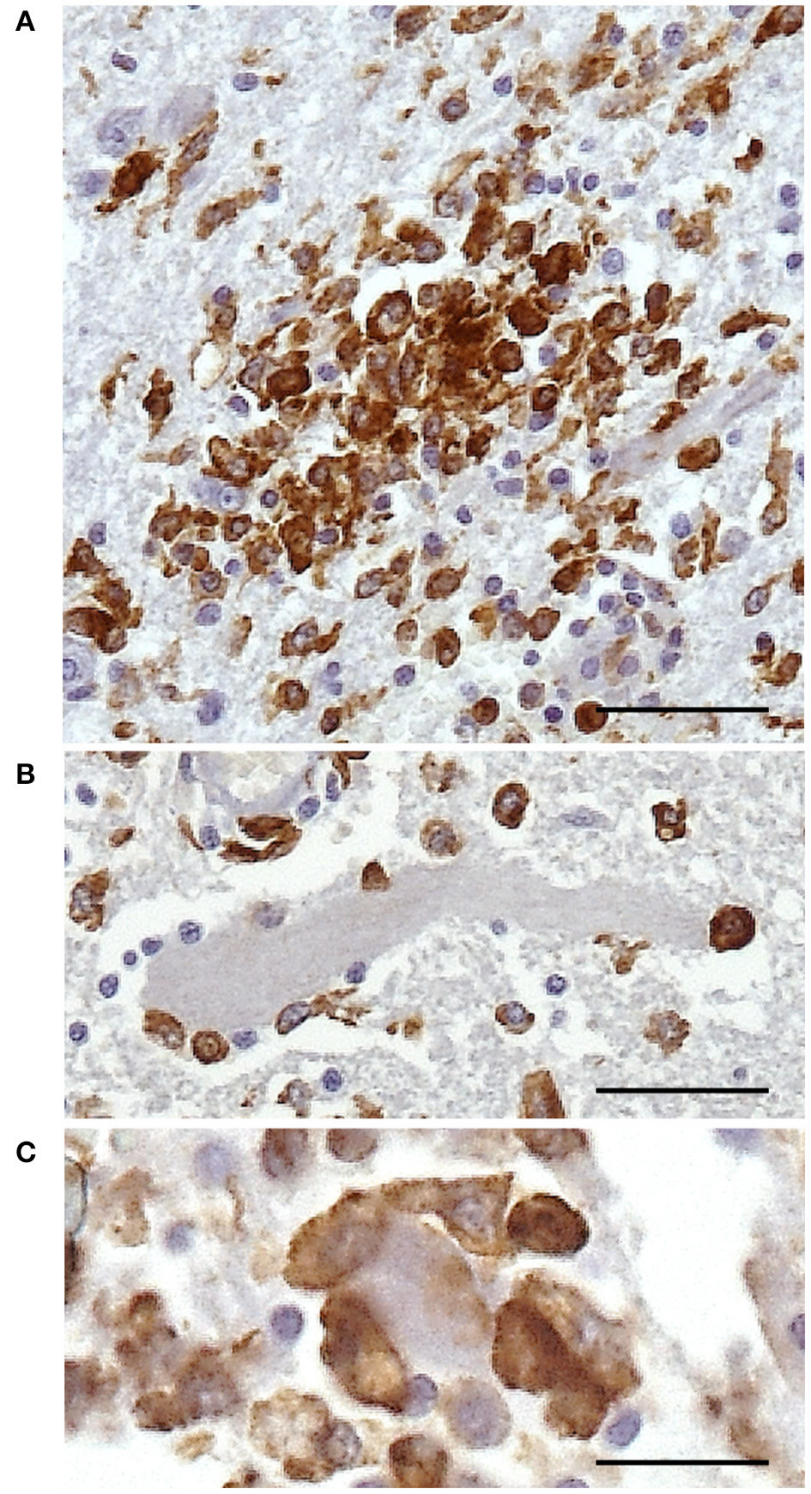
Immunohistochemical demonstration of Iba1 in the CNS of horses. Morphologic alterations of microglia in the spinal cord of horse 1. **(A)** Microglia nodule. **(B)** Iba1+ cells surrounding a neuron. **(C)** Neuronophagia is demonstrated by densely packed Iba1+ cells around and over a neuron. DAB, ×400 high-power field. Scale bar: 25 μm. Iba1, ionized calcium-binding adapter molecule 1; CNS, central nervous system.

Additionally, microglial nodules were present in 7/35 cases—five with a viral infection and two with parasitic infection (protozoa)—formed by large and compact groups of strongly stained cells (Figure 5A). Iba1+ cells were also demonstrated accompanying satellite cells around neurons and/or during neuronophagia in 18/35 cases (Figures 5B,C). These features were observed in 11/14 cases of viral infection, 1/4 case of an unknown pathogen, 5/6 cases of parasitic infection, and 1/6 cases of suggested bacterial toxic infection.

#### Determination of the Number of Ionized Calcium-Binding Adapter Molecule 1+ Cells in Different Lesion Setups

**Figure 6:**
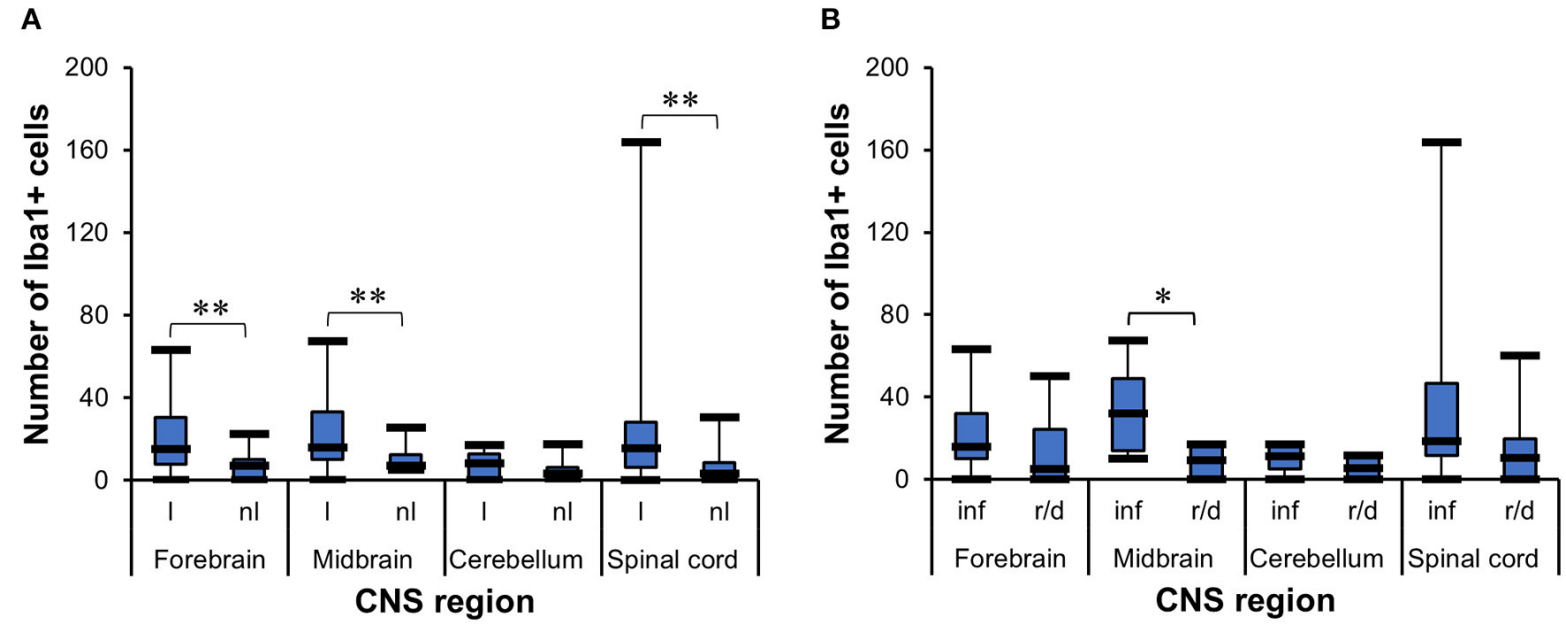
Analysis of Iba1 immunohistochemistry for microgliosis grade in the CNS of horses. **(A)** *t*-Test for dependent samples showed a significantly higher mean of Iba1+ cells in lesioned areas than non-lesioned areas from the forebrain, midbrain, and spinal cord. The number of stained cells was counted in five lesioned and five non-lesioned microscopic fields magnified ×200. **p ≤ 0.01. l, lesioned area; nl, non-lesioned area. **(B)** Wilcoxon–Mann–Whitney test demonstrated the statistically significant difference between the median number of stained Iba1+ cells in inflammatory lesions and reactive/degenerative lesions in five microscopic fields magnified at ×200 in the midbrain. *p ≤ 0.05. inf, inflammatory lesion; r/d, reactive and/or degenerative lesion; Iba1, ionized calcium-binding adapter molecule 1; CNS, central nervous system.

The *t*-test for dependent samples showed that there was a statistically significantly higher mean of Iba1+ cells in the lesioned areas from forebrain [*t*_(24)_ = 3.87, *p* = 0.007], midbrain [*t*_(13)_ = 3.31, *p* = 0.0056], and spinal cord [*t*_(21)_ = 3.02, *p* = 0.0065] than in non-lesioned areas, but there was no statistical difference in cerebellum [*t*_(11)_ = 1.25, *p* = 0.2365] (Figure 6A). The WMWT indicated that the median of Iba1+ cells was higher in inflammatory lesions (median = 32) than in degenerative lesions (median = 9.4) only in the midbrain (*U* = 39.5, *p* = 0.0230) (Figure 6B). From the pathogenetic view, this could indicate a shift toward lesions caused by viruses and parasites and a possible tropism for this brain area.

#### Comparison of Microglial Grades of Alteration in Different Lesion Setups

**Figure 7:**
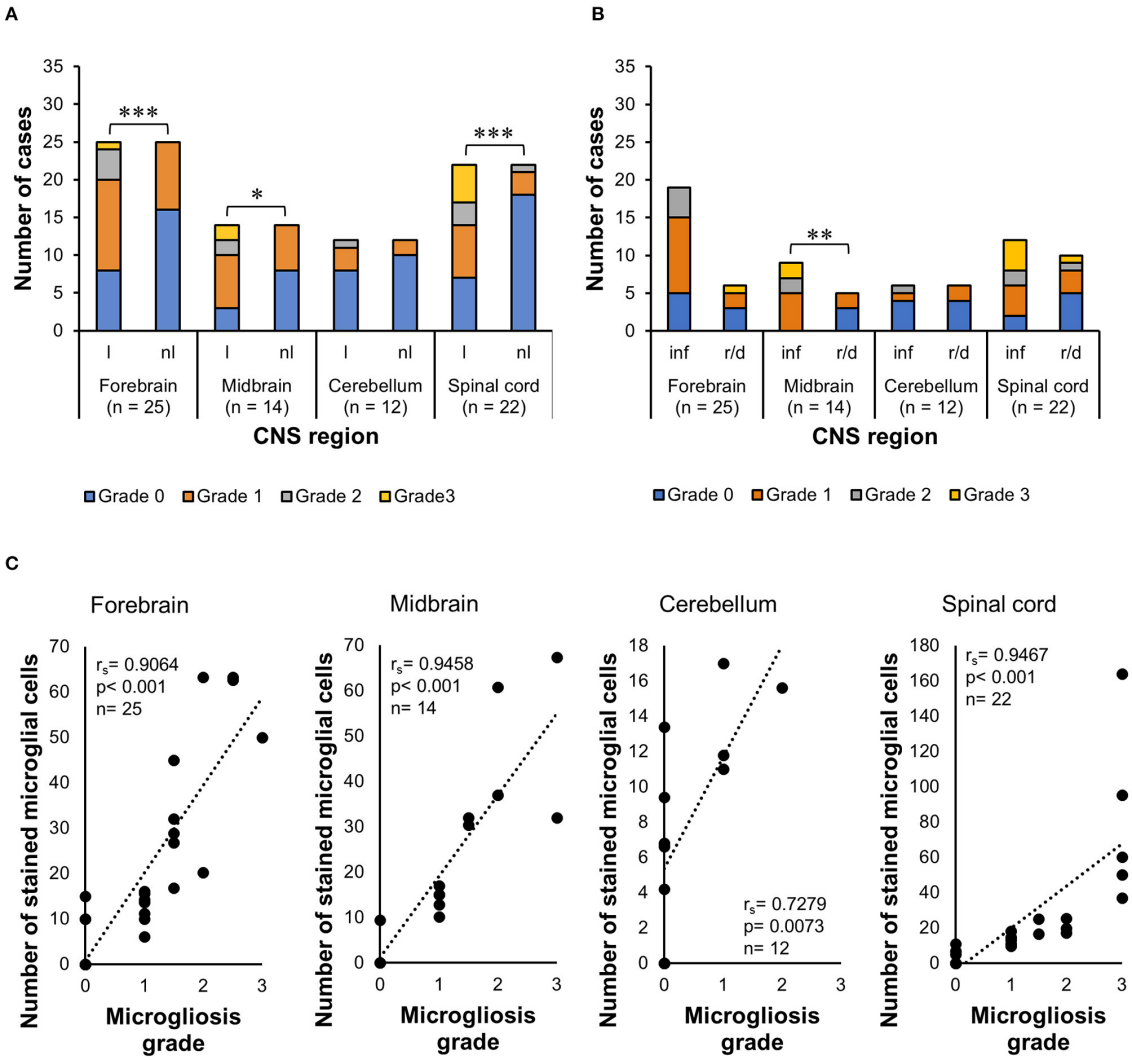
Analysis of Iba1 immunohistochemistry for microgliosis grade in the CNS of horses. **(A)** Wilcoxon signed-rank test demonstrated that there was also an increase in the grade of morphologic alterations in lesioned areas of the forebrain, midbrain, and spinal cord, compared with the non-lesioned areas in these regions. *p ≤ 0.05. ***p < 0.001. l, lesioned area; nl, non-lesioned area; n, number of CNS sections available. **(B)** Wilcoxon–Mann–Whitney test with exact inference showed that microglial alteration grades were statistically significantly higher in inflammatory lesions of the midbrain compared with reactive/degenerative lesions. **p < 0.01. *p ≤ 0.05. inf, inflammatory lesions; r/d, reactive and/or degenerative lesions. **(C)** Spearman's rank correlation coefficient showed the positive relationship between the increase in the mean of Iba1+ cells and the increase in microgliosis grade. r_s_, Spearman's correlation coefficient; Iba1, ionized calcium-binding adapter molecule 1; CNS, central nervous system.

The Wilcoxon signed-rank test indicated that lesioned areas also presented statistically significant higher morphologic alteration grades than non-lesioned areas (*p* < 0.001) in the midbrain (*p* = 0.0020) and spinal cord (*p* < 0.001), but not in the cerebellum (*p* = 0.5313) (Figure 7A). The WMWT with exact inference demonstrated that the microgliosis grade of inflammatory lesions in the midbrain was statistically significantly higher than that of degenerative lesions (*p* = 0.0070) (Figure 7B). In the spinal cord sections, the inflammatory lesions also showed higher activation grades, but this difference did not reach statistical significance. Spearman's rank correlation coefficient revealed that there was generally a positive relationship between the increase in the mean of Iba1+ cells and the increase in the grade of microglial activation in the lesioned areas of the CNS (Figure 7C). Mid-scale grades like 1.5 and 2.5 were also observed and could indicate continuous morphological changes in the tissue, with different stages of alteration in the same area.

## Discussion

Like humans, horses are susceptible to neurotropic and neuroinvasive pathogens that are not always readily identified in histological sections. Instead, alterations in astrocytes and microglia cells can be used as pathological hallmarks of injured nervous tissue in a variety of infectious and degenerative diseases (7). However, equine glial cell alterations are yet poorly characterized. Studies that demonstrate astrocytic and microglial activation in the CNS of horses have so far focused on describing morphological alterations associated with a known disease/alteration (2, 4).

Therefore, in this study, quantitative and morphological profiles of astrocytes and microglia of horses were translated into a scoring system, which was statistically verified using 35 cases of encephalitis/encephalopathies and four non-altered controls. This scoring system could also help to identify alterations undergone by glial cells when an etiology is undetermined, using the vastly used IHC markers GFAP and Iba1. Furthermore, recent studies with machine learning methods could benefit from this score, as it would provide a morphological template of different stages of alteration to complement quantification algorithms (12, 13).

### Immunohistochemical Demonstration of Equine Astrocytes

In this study, normal astrocytes (grade 0) appeared as staining in a range of 0 to 30 astrocytes/×200 magnification that were well-ramified with minimal contact between cells and tightly connected with blood vessels as also described in the spinal cord of horses by Meneses et al. (27) and in fibrous astrocytes from the cerebral cortex in control horses from Regina et al. (28). Studies in other animal species describe a similar morphology in non-altered CNS of dogs (29), mice (30), and rats (31), but cattle have a higher number of GFAP+ cells and non-altered astrocytes approximate morphologically to our grade 1 for horses (32). Limited or no proliferation of astrocytes was observed in mouse models of Alzheimer's disease and amyotrophic lateral sclerosis (33–35). In mild astrogliosis (grade 1), slight astrocytic morphologic alterations consisted of mild nuclear enlargement and an apparent increase in positive cells, ranging from 16 to 60/×200 magnification. These features were also described by Campos et al. (36) and de Sousa et al. (37) in the astrocytes from horses infected with the arbovirus Eastern equine encephalitis virus (EEEV) and match with the findings in this study in horses 24–27 and 32. There were mild alterations also in the rabies virus case (horse 2), with a comparable number and morphology of astrocytes found at the beginning of the infection demonstrated in the spinal cord of mice (38). Although there are no reports on grading from equine astrocytes in the literature, some authors described at least the increase in the number of GFAP+ cells in lesioned brain areas compared with normal horse brains (2, 4) or in studies about canine visceral leishmaniasis (39), canine distemper disease (40), and rhesus macaques with neurobrucellosis (41). Mild astrocytic activation was also observed in at least one non-altered region of the CNS of cases with degenerative lesions (like in horses 12, 14, 16, 17, and 28), associated with more severe alterations in other brain areas. This feature was already reported in neurotoxic events induced by mycotic, plant, and botulinum toxins, and uremic/hepatic encephalopathy (42–46). In moderate astrogliosis (grade 2), there were 40 to 100 GFAP+ cells/×200 magnification, accompanied by a more prominent cellular hypertrophy, and astrocytic processes overlapped. With this configuration, suggested parasitic infections like those displayed by horses 1, 9, 18, 19, 20, and 22 are supported by the findings demonstrated in mice experimentally infected with *Toxocara canis* (47, 48) and pigs with neurocysticercosis (49). On the other hand, Lemos and Alessi (28) found that astrocytes from horses suffering from leucoencephalomalacia displayed morphological alterations compatible with our grade 2, but with fewer GFAP+ cells. These findings justify the importance to address morphological and quantitative alterations of astrocytes in different pathological settings. Severe astrogliosis (grade 3) was associated with pronounced GFAP immunostaining and more than 77 GFAP+ astrocytes/×200 magnification with severe alterations. Like in severe human astrogliosis, there was pronounced cellular hypertrophy of the cell body and processes in horses. There was substantial intermingling and overlapping of neighboring astrocyte processes with blurring and disruption of individual astrocyte domains, as described by Sofroniew and Vinters (5). In the literature, this severe diffuse reaction pattern is described in severe focal lesions, infections, and chronic neurodegeneration (5). Comparably, all horses with grade 3 astrogliosis also had some degree of inflammatory lesions that were caused by a virus (horses 4, 24, 27, and 33) or a toxin (horse 11). Occasionally, gemistocytes were seen in the forebrain of horse 4 and midbrain of horse 29, which occur when there is lethal injury toward astrocytes, and in these cases, they were associated with arbovirus infections (16, 40). Upon a flaviviral infection, highly activated astrocytes increase their production of GFAP, which, combined with the release of inflammatory cytokines and chemokines by other components of the CNS, can lead to a severe course of the disease with a higher fatality rate (50, 51). Moreover, differences in protoplasmic and fibrous astrocytes were not explored at length since most of the lesions were observed in the gray matter. When present, lesions in the white matter were associated with cases of protozoan infections in the spinal cord (grade 2) and degenerative myelopathies (grade 2), similar to the cases of equine back pain from Mayaki et al. (52).

The statistical analysis carried out with the equine samples supported the morphological astrocytic alterations translated into the scoring system established. Correlation tests indicated that lesioned areas have a higher number (*p* ≤ 0.05) of GFAP+ astrocytes than non-lesioned areas and higher astrocytic alteration grades, independently of an inflammatory or degenerative lesion that triggered the response. The positive correlation obtained with a Spearman's rank correlation coefficient showed that the astrogliosis grades were correctly designated, as the higher the number of GFAP+ cells, the more severe were the morphological alterations.

The statistical model established for equine samples addressed some of the missing aspects regarding astrocytic reactions in horses. As observed in humans, astrogliosis in horses occurs in a spectrum of heterogeneous changes, translated into alteration grades (grades 1, 2, and 3) in this study. Besides, astrogliosis is widely used as a hallmark of diseased CNS tissue, thus the correct designation of morphologic and quantitative astrocytic alterations, which were missing for horses, can also help to relate certain pathogens/conditions in unresolved cases of encephalitis and encephalopathies.

### Immunohistochemical Demonstration of Equine Microglia Cells

Resting microglia (grade 0) were characterized by faintly stained cells that exhibited ramified and slender processes, with inconspicuous, small, hyperchromatic, and wedge-shaped nucleus, also observed by other authors (1). Resting microglia were found in all four non-altered equine brains used as controls, in non-lesioned areas bordering lesions, and in CNS regions completely lesion-free (but in cases with alterations in other sections). A similar morphology of resting microglia was described in early studies of microglia features (23), in mice (30), and the equine non-altered controls from an experimental study with *Trypanosoma evansi* infection (2). A mild microglial activation (grade 1) was characterized by a range of 6 to 30 Iba1+ cells/×200 magnification, and the morphological alterations were discrete, inclined to the beginning of cellular processes retraction. Grade 1 was the highest alteration observed in 17/35 cases, and although there are no reports of solely mild microgliosis as the main microglial alteration in horses, resting microglia are progressively activated in traumatic injuries, ischemic stroke, demyelination, and neurodegenerative diseases (53). In the four cases described as flaviviral infection (horses 3 to 6), this pattern was also observed and could indicate a delay in the immunological answer of microglia cells toward these pathogens (54). Flaviviruses, among other viruses, might invade the CNS through Trojan horse mechanisms, remain undetected by the host immune system for a longer period, and cause further neurological alterations (54). In other 4/35 cases (horses 10, 24, 26, and 27), this mild activation was associated with more severe grades in other CNS regions. In the moderate level of activation (grade 2) observed in 5/35 cases (horses 15, 23, 24, 26, and 29), a range of 17 to 60 cells/×200 magnification were demonstrated with distinguishable cellular processes retraction, and a general hypertrophic reaction with an increased number of Iba1+ cells, also described by other studies (16, 53, 55). Grade 3 microglia activation was observed in 7/35 cases and was associated with Gitter cell occurrence. These bulky cells, peripherally stained with Iba1, were observed concurrently with other smaller, densely packed, and intensely stained microglia. These activated smaller cells were also seen within vessels, a sign of peripheral migration of monocytes as reported in severe microgliosis associated with blood–brain barrier damage in experimental autoimmune encephalomyelitis (14). Furthermore, Gitter cells were observed within brain areas of horses displaying inflammatory lesions caused by parasites (horses 1, 9, and 22), viruses (horses 7 and 27), and toxins (horse 10) and in a case with degenerative spinal cord of unknown origin (horse 8). Other studies that assessed equine microglia demonstrated these highly activated cells in lesions produced in equine protozoan myeloencephalitis (2, 56, 57), by *Halicephalobus gingivalis* (58), rabies virus (59), *Trema micrantha* intoxication (60), and leucoencephalomalacia by mycotoxicosis (44). Furthermore, moderate-to-severe microgliosis was statistically associated with inflammation in the midbrain, with a tendency to occur in viral (horses 24, 27, and 29) and parasitic infections (horse 22). This feature could indicate a tropism from the pathogen to this brain area.

The statistical analysis also supported the morphological aspects attributed to the scoring system. Correlation tests indicated that lesioned areas from the forebrain, midbrain, and spinal cord have a higher number (*p* ≤ 0.05) of Iba1+ cells than non-lesioned areas, as well as higher microglial activation grade. Cerebellum sections were underrepresented in the study, which could have influenced the statistical analysis's lack of correlation. The positive correlation obtained with a Spearman's rank correlation coefficient showed that the microglia activation grades were correctly designated, as the higher the number of Iba1+ cells, the more severe were the morphological alterations.

Other interesting features of microglial activation in the CNS of horses and that could help assess an etiology underlying the alterations observed are microglial nodules; observed in 7/35 cases that presented non-suppurative encephalitis. It is still debatable if different pathogens (viruses vs. parasites) induce different glial reaction patterns. However, we confirmed in our study the predominance of microglial nodule formation by viral infections in horses (in 5/7 cases) (4, 59, 61, 62). For the other two cases (horses 1 and 9), a protozoal infection was detected, and the nodule formation could indicate the persistence of neurophagic sites (63). Furthermore, neuronophagia was commonly observed among the cases studied (18/35), with no predominance for an activation grade. However, there was a strong tendency to occur in samples with inflammation, as a feature of many viral infections (16). Additionally, reactive microglia also develop into rod cells, a bipolar ramified configuration. Again, these are most commonly seen in neurotropic viral diseases, like Borna disease in horses (16), but are also reported in human cases of subacute sclerosing panencephalitis, Alzheimer's disease, and Wilson's disease (64) and in horses experimentally infected with *T. evansi* (2). Although their function is not entirely understood, they are observed when there is diffuse brain injury (11, 65), going along with the extensive and severe lesions observed in tissues from horses 1 and 22 in this study.

## Conclusions

The statistical model established in this study addressed some of the missing aspects regarding astrocytic and microglial reactions in horses. Like observed in humans, these alterations occur in a spectrum of heterogeneous changes, here translated into grades 1, 2, and 3. Also, astrogliosis and microgliosis are widely used as a hallmark of diseased CNS tissue, and therefore, the correct designation of morphologic and quantitative alterations, which were missing for horses, can also help to relate certain etiologies in unresolved cases of encephalitis and encephalopathies. Moreover, this study can serve as a template for studies in segments of artificial intelligence to quantify glial cells. On the other hand, the lack of complete CNS sets in all cases can be a limitation if a profound assessment of the alterations undergone by each etiology is sought. However, on a routine basis in diagnostics, pathologists hardly have all CNS areas available, and the center of interest for investigation is sections with lesions, which were well-represented in the study.

## Data Availability Statement

The raw data supporting the conclusions of this article will be made available by the authors, without undue reservation.

## Ethics Statement

Ethical review and approval was not required for the animal study because this work was carried out with archived material formalin-fixed paraffin-embedded. Written informed consent for participation was not obtained from the owners because this work was carried out with archived material formalin-fixed paraffin-embedded. There is no mention on names, specific places, or any other form that would allow the identification of owners and animals.

## Author Contributions

GB contributed to the conception, designed the study, and organized and wrote the manuscript. KF performed the statistical analysis. EC, DD, MC, DB, and JD collected the samples and provided clinical and histological reports. CH contributed to the conception, design of the study, and manuscript writing. All authors revised the manuscript revision and read and approved the submitted version.

## Conflict of Interest

The authors declare that the research was conducted in the absence of any commercial or financial relationships that could be construed as a potential conflict of interest.

## References

[1] Delcambre GH Liu J Herrington JM Vallario K Long MT. Immunohistochemistry for the detection of neural and inflammatory cells in equine brain tissue. PeerJ. (2016) 4:e1601. 10.7717/peerj.1601 26855862 PMC4741088

[2] Lemos KR Marques LC Aquino LPCT Alessi AC Zacarias RZ. Astrocytic and microglial response and histopathological changes in the brain of horses with experimental chronic Trypanosoma evansi infection. Rev Inst Med Trop São Paulo. (2008) 50:243–9. 10.1590/S0036-46652008000400011 18813766

[3] Salouci M Antoine N Shikh Al Sook MK Piret J Mignon Y Kirschvink N. Developmental profiles of GFAP-positive astrocytes in sheep cerebellum. Vet Res Commun. (2014) 38:279–85. 10.1007/s11259-014-9614-1 25113608

[4] Delcambre GH Liu J Streit WJ Shaw GPJ Vallario K Herrington J. Phenotypic characterisation of cell populations in the brains of horses experimentally infected with West Nile virus. Equine Vet J. (2017) 49:815–20. 10.1111/evj.12697 28470955

[5] Sofroniew MV Vinters HV. Astrocytes: biology and pathology. Acta Neuropathol. (2010) 119:7–35. 10.1007/s00401-009-0619-8 20012068 PMC2799634

[6] Pekny M Pekna M. Astrocyte reactivity and reactive astrogliosis: costs and benefits. Physiol Rev. (2014) 94:1077–98. 10.1152/physrev.00041.2013 25287860

[7] Kovacs GG. Cellular reactions of the central nervous system. In: Kovacs GG Alafuzoff I editors. Handbook of Clinical Neurology. 1st ed. Amsterdan: Elsevier B.V. (2018). p. 13–23. 10.1016/B978-0-12-802395-2.00003-1 28987163

[8] Sofroniew MV. Molecular dissection of reactive astrogliosis and glial scar. Trens Neurosci. (2009) 32:638–47. 10.1016/j.tins.2009.08.002 19782411 PMC2787735

[9] Shapiro LA Perez ZD Foresti ML Arisi GM Ribak CE. Morphological and ultrastructural features of Iba1-immunolabeled microglial cells in the hippocampal dentate gyrus. Brain Res. (2009) 1266:29–36. 10.1016/j.brainres.2009.02.031 19249294 PMC2677570

[10] Ziebell JM Taylor SE Cao T Harrison JL Lifshitz J. Rod microglia: elongation, alignment, and coupling to form trains across the somatosensory cortex after experimental diffuse brain injury. J Neuroinflamm. (2012) 9:247. 10.1186/1742-2094-9-247 23111107 PMC3526458

[11] Taylor SE Morganti-Kossmann C Lifshitz J Ziebell JM. Rod microglia: a morphological definition. PLoS ONE. (2014) 9:e97096. 10.1371/journal.pone.0097096 24830807 PMC4022629

[12] Liu M Ylanko J Weekman E Beckett T Andrews D McLaurin J. Utilizing supervised machine learning to identify microglia and astrocytes *in situ*: implications for large-scale image analysis and quantification. J Neurosci Methods. (2019) 328:108424. 10.1016/j.jneumeth.2019.108424 31494186

[13] Healy S McMahon J FitzGerald U. Seeing the wood for the trees: towards improved quantification of glial cells in central nervous system tissue. Neural Regen Res. (2018) 13:1520. 10.4103/1673-5374.235222 30127105 PMC6126125

[14] Ajami B Bennett JL Krieger C McNagny KM Rossi FM V. Infiltrating monocytes trigger EAE progression, but do not contribute to the resident microglia pool. Nat Neurosci. (2011) 14:1142–9. 10.1038/nn.2887 21804537

[15] Serrano-Pozo A Gómez-Isla T Growdon JH Frosch MP Hyman BT. A phenotypic change but not proliferation underlies glial responses in Alzheimer disease. Am J Pathol. (2013) 182:2332–44. 10.1016/j.ajpath.2013.02.031 23602650 PMC3668030

[16] Cantile C Youssef S. Nervous system. In: Maxie G editor. Jubb, Kennedy & Palmer's Pathology of Domestic Animals. Sixth. Saint Louis, MO: Elsevier (2016). p. 250–406.

[17] Angenvoort J Brault AC Bowen RA Groschup MH. West Nile viral infection of equids. Vet Microbiol. (2013) 167:168–80. 10.1016/j.vetmic.2013.08.013 24035480 PMC4581842

[18] Bender JB Tsukayama DT. Horses and the risk of zoonotic infections. Vet Clin North Am - Equine Pract. (2004) 20:643–53. 10.1016/j.cveq.2004.07.003 15519824 PMC7118998

[19] Kumar B Manuja A Gulati B Virmani N Tripathi BN. Zoonotic viral diseases of equines and their impact on human and animal health. Open Virol J. (2018) 12:80–98. 10.2174/1874357901812010080 30288197 PMC6142672

[20] Meneses CS Müller HY Herzberg DE Uberti B Werner MP Bustamante HA. Microglia and astrocyte activation in the spinal cord of lame horses. Vet Anaesth Analg. (2018) 45:92–102. 10.1016/j.vaa.2017.10.001 29223561

[21] Boos GS. Non-suppurative Encephalitis and Encephalopathies of Unknown Origin in Horses From Brazil. Justus-Liebig-University, Giessen (2020).

[22] Kaufmann W Bolon B Bradley A Butt M Czasch S Garman RH. Proliferative and nonproliferative lesions of the rat and mouse central and peripheral nervous systems. Toxicol Pathol. (2012) 40:87–157S. 10.1177/0192623312439125 22637737

[23] Kreutzberg GW. Microglia: a sensor for pathological events in the CNS. Trends Neurosci. (1996) 19:312–8. 10.1016/0166-2236(96)10049-7 8843599

[24] Lemstra AW Groen in't Woud JCM Hoozemans JJM van Haastert ES Rozemuller AJM Eikelenboom P. Microglia activation in sepsis: a case-control study. J Neuroinflammation. (2007) 4:4. 10.1186/1742-2094-4-4 17224051 PMC1783646

[25] Dixon WJ. BMDP Statistical Software Manual 1992: BMDP Release 7 v.1. Los Angeles, CA: University of California Press (1993). p. 698.

[26] CYTEL Inc. Cytel Studio StatXact Version 9.0.0. In: CYTEL Software Corporation editor. Statistical Software for Exact Nonparametric Inference, User Manual. Cambridge, MA: CYTEL Inc. (2010).

[27] Meneses CS Müller HY Herzberg DE Uberti B Bustamante HA Werner MP. Immunofluorescence characterization of spinal cord dorsal horn microglia and astrocytes in horses. PeerJ. (2017) 5:e3965. 10.7717/peerj.3965 29085760 PMC5661433

[28] Regina K Carlos A R ALK Glial AAC Veterinária P Depto B. Astrócitos imunorreativos à proteína glial fibrilar ácida (GFAP) em sistema nervoso central de equinos normais e de equinos com leucoencefalomalácia. Pesqui Vet Bras. (1999) 19:104–8. 10.1590/S0100-736X1999000300003

[29] Hwang IK Choi JH Li H Yoo K-Y Kim DW Lee CH. Changes in glial fibrillary acidic protein immunoreactivity in the detate gyrus and hippocampus proper of adult and aged dogs. J Vet Med Sci. (2008) 70:965–9. 10.1292/jvms.70.965 18840972

[30] Kim JW Nam SM Yoo DY Jung HY Hwang IK Seong JK. Strain-specific differential expression of astrocytes and microglia in the mouse hippocampus. Brain Behav. (2018) 8:e00961. 10.1002/brb3.961 29761014 PMC5943717

[31] Kálmán M Hajós F. Distribution of glial fibrillary acidic protein (GFAP)-immunoreactive astrocytes in the rat brain - I. Forebrain. Exp Brain Res. (1989) 78:147–63. 10.1007/BF00230694 2591509

[32] Machado GF Alessi AC. Astrócitos imunorreativos à proteína glial fibrilar ácida (GFAP) em SNC de bovinos normais e de bovinos com raiva. I. Hipocampo e giro dentato. Brazilian J Vet Res Anim Sci. (1997) 34:345. 10.11606/issn.2318-3659.v34i6p345-348

[33] Kamphuis W Orre M Kooijman L Dahmen M Hol EM. Differential cell proliferation in the cortex of the APPswePS1dE9 Alzheimer's disease mouse model. Glia. (2012) 60:615–29. 10.1002/glia.22295 22262260

[34] Sirko S Behrendt G Johansson PA Tripathi P Costa MR Bek S. Reactive glia in the injured brain acquire stem cell properties in response to sonic hedgehog. Cell Stem Cell. (2013) 12:426–39. 10.1016/j.stem.2013.01.019 23561443

[35] Lepore AC Dejea C Carmen J Rauck B Kerr DA Sofroniew MV. Selective ablation of proliferating astrocytes does not affect disease outcome in either acute or chronic models of motor neuron degeneration. Exp Neurol. (2008) 211:423–32. 10.1016/j.expneurol.2008.02.020 18410928 PMC9152669

[36] Campos KF Oliveira CHS De Reis AB Yamasaki EM Brito MF Andrade SJT. Surto de encefalomielite equina Leste na Ilha de Marajó, Pará1. Pesqui Vet Bras. (2013) 33:443–8. 10.1590/S0100-736X2013000400005

[37] de Sousa SKH Sonne L Sant'Ana FJF de Junior JLR. Encefalomielite equina do leste no Distrito Federal e entorno. Acta Sci Vet. (2015) 43:1–6. Available online at: http://www.ufrgs.br/actavet/43/PUB%201268.pdf

[38] Kojima D Park C-H Satoh Y Inoue S Noguchi A Oyamad T. Pathology of the spinal cord of C57BL/6J mice infected with rabies virus (CVS-11 strain). J Vet Med Sci. (2009) 71:319–24. 10.1292/jvms.71.319 19346700

[39] Melo GD Machado GF. Glial reactivity in dogs with visceral leishmaniasis: correlation with T lymphocyte infiltration and with cerebrospinal fluid anti-Leishmania antibody titres. Cell Tissue Res. (2011) 346:293–304. 10.1007/s00441-011-1290-7 22160561

[40] Headley SA Soares IC Graça DL. Glial Fibrillary Acidic Protein (GFAP)-immunoreactive astrocytes in dogs infected with canine distemper virus. J Comp Pathol. (2001) 125:90–7. 10.1053/jcpa.2001.0483 11578123

[41] Lee KM Chiu KB Sansing HA Didier PJ Ficht TA Arenas-Gamboa AM. Aerosol-induced brucellosis increases TLR-2 expression and increased complexity in the microanatomy of astroglia in rhesus macaques. Front Cell Infect Microbiol. (2013) 3:86. 10.3389/fcimb.2013.00086 24350061 PMC3844859

[42] Bouchard PR Weldon AD Lewis RM Summers BA. Uremic encephalopathy in a horse. Vet Pathol. (1994) 31:111–5. 10.1177/030098589403100116 8140716

[43] Frye MA Johnson JS Traub-Dargatz JL Savage CJ Fettman MJ Gould DH. Putative uremic encephalopathy in horses: five cases (1978-1998). J Am Vet Med Assoc. (2001) 218:560–6. 10.2460/javma.2001.218.560 11229510

[44] Giannitti F Diab SS Pacin AM Barrandeguy M Larrere C Ortega J. Equine leukoencephalomalacia (ELEM) due to fumonisins B1 and B2 in Argentina. Pesqui Vet Bras. (2011) 31:407–12. 10.1590/S0100-736X2011000500007

[45] Bandarra PM Pavarini SP Raymundo DL Correa AMR Pedroso PMO Driemeier D. Trema micrantha toxicity in horses in Brazil. Equine Vet J. (2010) 42:456–9. 10.1111/j.2042-3306.2010.00035.x 20636784

[46] Jenkinson SP Grandgirard D Heidemann M Tscherter A Avondet M-A Leib SL. Embryonic stem cell-derived neurons grown on multi-electrode arrays as a novel *in vitro* bioassay for the detection of *Clostridium botulinum* neurotoxins. Front Pharmacol. (2017) 8:73. 10.3389/fphar.2017.00073 28280466 PMC5322221

[47] Eid MM El-Kowrany SI Othman AA El Gendy DI Saied EM. Immunopathological changes in the brain of immunosuppressed mice experimentally infected with Toxocara canis. Korean J Parasitol. (2015) 53:51–8. 10.3347/kjp.2015.53.1.51 25748709 PMC4384791

[48] Liao C-W Cho W-L Kao T-C Su K-E Lin Y-H Fan C-K. Blood-brain barrier impairment with enhanced SP, NK-1R, GFAP and claudin-5 expressions in experimental cerebral toxocariasis. Parasite Immunol. (2008) 30:525–34. 10.1111/j.1365-3024.2008.01048.x 18627507

[49] Sikasunge CS Johansen M V. Phiri IK Willingham AL Leifsson PS. The immune response in Taenia solium neurocysticercosis in pigs is associated with astrogliosis, axonal degeneration and altered blood-brain barrier permeability. Vet Parasitol. (2009) 160:242–50. 10.1016/j.vetpar.2008.11.015 19117683

[50] Bardina S V Lim JK. The role of chemokines in the pathogenesis of neurotropic flaviviruses. Immunol Res. (2012) 54:121–32. 10.1007/s12026-012-8333-3 22547394 PMC12947276

[51] Pekny M Pekna M. Reactive gliosis in the pathogenesis of CNS diseases. Biochim Biophys Acta Mol Basis Dis. (2016) 1862:483–91. 10.1016/j.bbadis.2015.11.014 26655603

[52] Mayaki AM Abdul Razak IS Mohd Adzahan N Mazlan M Abdullah R. Myelopathy and reactive microgliosis and astrogliosis in equine back pain. J Equine Vet Sci. (2020) 90:103019. 10.1016/j.jevs.2020.103019 32534783

[53] Li T Zhang S. Microgliosis in the injured brain: infiltrating cells and reactive microglia both play a role. Neurosci. (2015) 22:165–70. 10.1177/1073858415572079 25672621

[54] Mustafá YM Meuren LM Coelho SVA de Arruda LB. Pathways exploited by flaviviruses to counteract the blood-brain barrier and invade the central nervous system. Front Microbiol. (2019) 10:28. 10.3389/fmicb.2019.00525 30984122 PMC6447710

[55] Cartier N Lewis CA Zhang R Rossi FM V. The role of microglia in human disease: therapeutic tool or target? Acta Neuropathol. (2014) 128:363–80. 10.1007/s00401-014-1330-y 25107477 PMC4131134

[56] MacKay RJ Granstrom DE Saville WJ Reed SM. Equine protozoal myeloencephalitis. Vet Clin North Am Equine Pract. (2000) 16:405–25. 10.1016/S0749-0739(17)30086-X 11219340

[57] Witonsky S Sellon DC Dubey JP. Equine protozoal myeloencephalitis. In: Sellon DC Long MT editors. Equine Infectious Diseases. 2nd ed. Saint Louis, MO: Elsevier (2014) p. 456–67.e6.

[58] Vasconcelos RDO Lemos KR De Moraes JRE Borges VP. Halicephalobus gingivalis (H.deletrix) in the brain of a horse. Cienc Rural. (2007) 37:1185–7. 10.1590/S0103-84782007000400047

[59] Bassuino D Konradt G Cruz RAS Silva GS Gomes DC Pavarini SP. Characterization of spinal cord lesions in cattle and horses with rabies. J Vet Diagn Investig. (2016) 28:455–60. 10.1177/1040638716647992 27240569

[60] Lorenzett MP Pereira PR Bassuino DM Konradt G Panziera W Bianchi M V. Neurotoxicosis in horses associated with consumption of Trema micrantha. Equine Vet J. (2018) 50:192–5. 10.1111/evj.12741 28805273

[61] Bielefeldt-Ohmann H Bosco-Lauth A Hartwig A-E Uddin MJ Barcelon J Suen WW. Characterization of non-lethal West Nile Virus (WNV) infection in horses: subclinical pathology and innate immune response. Microb Pathog. (2017) 103:71–9. 10.1016/j.micpath.2016.12.018 28012987

[62] Cantile C Piero F del di Guardo G Arispici M. Pathologic and immunohistochemical findings in naturally occurring west nile virus infection in horses. Vet Pathol. (2001) 38:414–21. 10.1354/vp.38-4-414 11467475

[63] Eydal M Bambir SH Sigurdarson S Gunnarsson E Svansson V Fridriksson S. Fatal infection in two Icelandic stallions caused by *Halicephalobus gingivalis* (Nematoda: Rhabditida). Vet Parasitol. (2012) 186:523–7. 10.1016/j.vetpar.2011.11.024 22305655

[64] Wierzba-Bobrowicz T Gwiazda E Kosno-Kruszewska E Lewandowska E Lechowicz W Bertrand E. Morphological analysis of active microglia–rod and ramified microglia in human brains affected by some neurological diseases (SSPE, Alzheimer's disease and Wilson's disease). Folia Neuropathol. (2002) 40:125–31. 12572918

[65] Fumagalli S Perego C Pischiutta F Zanier ER De Simoni M-G. The ischemic environment drives microglia and macrophage function. Front Neurol. (2015) 6:81. 10.3389/fneur.2015.00081 25904895 PMC4389404

