# Supplementary material for: Glial Fibrillary Acidic Protein and Ionized Calcium-Binding Adapter Molecule 1 Immunostaining Score for the Central Nervous System of Horses With Non-suppurative Encephalitis and Encephalopathies
Source: Front Vet Sci. 2021 Jul 9;8:660022. doi: 10.3389/fvets.2021.660022 (PMC8299206; doi:10.3389/fvets.2021.660022)
Supplement: Supplementary file 1 [file Table_1.docx]

**Supplementary material 1**: **Cases used to conduct the study of astrocytic and microglial alterations from the central nervous system of horses**

| **ID** | **Age** | **Location** | **Year/ Season** | **Material available** | **Lesion type** | **Etiology** | **Gemisto- cytes** | **Mean astrocytes^a^** | **GFAP grade** | **Microglia node** | **Neuron.** | **Mean microglia^a^** | **Iba1 grade** |
| --- | --- | --- | --- | --- | --- | --- | --- | --- | --- | --- | --- | --- | --- |
| 1 | 6 years | Midwest | 2008  Summer | Spinal cord | Inflammatory | *S. neurona* | - | 28.4 | 2 | + | + | 163.8 | 3 |
| 2 | 8 years | Midwest | 2008  Summer | Spinal cord | Inflammatory | Rabies virus | - | 16.8 | 1 | - | - | 14.6 | 1 |
| 3 | 3 years | Midwest | 2009  Summer | Cerebral cortex | Inflammatory | Flavivirus | - | 49.6 | 1.5 | - | + | 26.8 | 1 |
| 4 | 7 years | Midwest | 2009  Summer | Cerebellum,  Cerebral cortex | Inflammatory | Flavivirus | -  + | 36.2  100 | 1  3 | -  - | -  + | 0  14.2 | 0  1 |
| 5 | 8 years | Midwest | 2009  Summer | Cerebellum,  Cerebral cortex | Inflammatory | Flavivirus | -  - | 54.6  53.6 | 1.5  2 | -  - | -  + | 13.4  13.6 | 0  1 |
| 6 | 10 years | Midwest | 2009  Summer | Cerebellum,  Cerebral cortex | Inflammatory | Flavivirus | -  - | 36.6  46.2 | 1  2.5 | -  - | -  + | 4.2  6 | 0  1 |
| 7 | 4 years | Midwest | 2009  Fall | Spinal cord,  Cerebral cortex,  Trig. ganglion | Inflammatory | Unknown pathogen | -  -  - | 28.6  64.4  * | 2  2  * | -  -  * | +  -  - | 17  50  * | 2  3  * |
| 8 | 2.5 years | South | 2013  Fall | Spinal cord,  Cerebellum,  Cerebral cortex | Degenerative | Trauma | -  -  - | 47.8  45.6  47.8 | 2.5  2.5  2.5 | -  -  - | -  -  - | 60.2  0  0 | 3  0  0 |
| 9 | N.a. | South | 2013  Fall | Spinal cord,  Nerve | Inflammatory | *S. neurona* | -  - | 46.4  * | 2.5  * | +  * | +  + | 95.2  * | 3  * |
| 10 | 6 years | South | 2013  Fall | Spinal cord,  Cerebellum,  Cerebral cortex | Inflammatory | Toxic-infection | -  -  - | 28.4  26  15.6 | 1  1  1 | -  -  - | -  -  - | 50  11.8  0 | 3  1  0 |
| 11 | 1.5 year | South | 2013  Fall | Cerebellum,  Cerebral cortex | Inflammatory | Toxic-infection | -  - | 91.4  37.8 | 3  1.5 | -  - | -  - | 0  0 | 0  0 |
| 12 | 6 years | South | 2013  Fall | Spinal cord,  Cerebral cortex | Degenerative | Toxic-infection | -  - | 25.2  23.2 | 1  1 | -  - | -  - | 0  0 | 0  0 |
| 13 | 7 years | South | 2013  Fall | Spinal cord,  Cerebellum,  Cerebral cortex | Degenerative | Toxic-infection | -  -  - | 45.8  65.8  44.6 | 2  2  2 | -  -  - | -  -  - | 9.6  6.6  0 | 1  0  0 |
| 14 | 2 years | South | 2013  Fal | Spinal cord,  Obex | Degenerative | Toxic-infection | -  - | 63.8  24.8 | 2  1 | -  - | -  - | 0  0 | 0  0 |
| 15 | 7 years | South | 2013  Fall | Spinal cord | Degenerative | Inconclusive | - | 41.4 | 2 | - | - | 25.4 | 2 |
| 16 | 16 years | South | 2013  Spring | Spinal cord,  Trig. ganglion  Obex,  Striatum | Inflammatory (only in trig. ganglion) | Ageing | -  -  -  - | 75  *  59.8  83.2 | 1.5  *  1.5  2 | -  *  -  - | -  -  -  - | 0  *  9.4  10 | 0  *  0  1 |
| 17 | 4 years | South | 2014  Fall | Spinal cord,  Cerebral cortex | Degenerative | Toxic-infection | -  - | 53.6  43.6 | 1.5  1.5 | -  - | -  + | 0  16.8 | 0  1 |
| 18 | 6 years | South | 2014  Fall | Spinal cord,  Mesencephalon | Inflammatory | *S. neurona* | -  - | 76.6  77.8 | 2.5  2.5 | -  - | +  + | 25  10.2 | 1  1 |
| 19 | 6 years | South | 2014  Fall | Spinal cord,  Cerebral cortex | Inflammatory | *S. neurona* | -  - | 0  35.6 | 0  2 | -  - | -  - | 5  16 | 0  1 |
| 20 | 7 years | South | 2014  Spring | Spinal cord,  Frontal cortex,  Parietal cortex,  Bulb | Inflammatory | *S. neurona* | -  -  -  - | 47  42.4  42.4  34 | 2  2  2  2 | -  -  -  - | +  -  -  - | 16.4  28.8  28.8  17 | 1  1.5  1.5  1 |
| 21 | 6 years | South | 2014  Spring | Spinal cord,  Trig. ganglion | Inflammatory (only in trig. ganglion) | Trauma | -  - | 46.6  * | 1.5  * | -  * | -  + | 0  * | 0  * |
| 22 | 10 years | Midwest | 2011  Early winter | Spinal cord,  Hippocampus,  Third ventricle,  Choroid plexus | Inflammatory | Undefined parasite | -  -  -  - | 40.2  42.2  52.6  * | 1.5  2  2.5  * | -  -  -  - | +  +  -  - | 37  20.2  32  * | 3  2  3  * |
| 23 | N.a. | Midwest | 2007 | Spinal cord | Inflammatory | Unknown pathogen | - | 42.2 | 1.5 | - | - | 19.8 | 2 |
| 24 | 6 months | North | Fall | Spinal cord,  Cerebellum,  Cerebral cortex,  Obex | Inflammatory | Alphavirus | -  -  -  - | 72.4  47.6  130.8  70.4 | 2  2  3  2 | +  -  -  - | +  -  -  + | 11  15.6  63.2  60.8 | 1  2  2  2 |
| 25 | 1.5 year | North | Fall | Cerebellum,  Choroid plexus,  Cerebral cortex,  Hippocampus,  C. callosum,  Spinal cord,  Bulb | Inflammatory | Alphavirus | -  -  -  -  -  -  - | 58  *  86.2  86.2  86.2  86  96.8 | 1.5  *  2.5  2.5  2.5  2  2.5 | -  -  -  -  -  -  - | -  -  -  +  +  -  - | 6.8  *  11.2  11.2  11.2  6.8  12.8 | 0  *  1  1  1  0  1 |
| 26 | 5 years | North | Fall | Hippocampus,  Cerebellum,  Mesencephalon | Inflammatory | Alphavirus | -  -  - | 56.2  101.6  44.8 | 1.5  2  2 | +  -  - | -  -  - | 63.2  9.4  32 | 2.5  0  1.5 |
| 27 | 4 years | North | Fall | Hippocampus,  Pons,  Spinal cord,  Cerebral cortex | Inflammatory | Alphavirus | -  -  -  - | 113.6  27.4  16.8  113.6 | 3  1.5  1  3 | -  -  +  - | -  -  -  - | 45  67.4  18  45 | 1.5  3  1  1.5 |
| 28 | N.a. | North | Fall | Spinal cord,  Cerebral cortex,  Hippocampus,  Cerebellum | Degenerative | Unknown pathogen | -  -  -  - | 72.4  79.4  79.4  74.6 | 1.5  1.5  1.5  2 | -  -  -  - | -  -  -  - | 11  15.6  15.6  11 | 0  1  1  1 |
| 29 | Adult | Midwest | 2005  Fall | Forebrain  Midbrain | Inflammatory | Alphavirus | -  + | 53  115 | 2.5  2.5 | +  + | +  + | 62.6  37 | 2  2 |
| 30 | Adult | Midwest | 2006 | Pons,  Mesencephalon | Inflammatory | Alphavirus | -  - | 69  69 | 2  2 | -  - | +  + | 30.4  30.4 | 1  1 |
| 31 | N.a. | Northeast | 2009 | Mesencephalon | Inflammatory | Alphavirus | - | 83.8 | 2 | - | + | 7 | 1 |
| 32 | 6 months | Midwest | 2011  Fall | Spinal cord,  Cerebral cortex,  Thalamus | Inflammatory | Alphavirus | -  -  - | 61.4  47.2  47.2 | 2  1.5  1.5 | +  -  - | +  +  - | 13  15  15 | 1  0  0 |
| 33 | 5 years | Midwest | 2012  Fall | Parietal cortex,  Thalamus | Inflammatory | Alphavirus | -  - | 195.4  195.4 | 3  3 | -  - | +  - | 32  32 | 1  1 |
| 34 | N.a. | Northeast | 2013 | Cerebral cortex | Inflammatory | Unknown pathogen | - | 48.6 | 1.5 | - | - | 10 | 0 |
| 35 | N.a. | Northeast | 2013 | Cerebral cortex | Inflammatory | Unknown pathogen | - | 81.4 | 2.5 | - | - | 10 | 0 |
|  |  |  |  | **Non-altered controls** |  |  |  |  |  |  |  |  |  |
| 36 | Adult | Germany | 2015 | Cerebral cortex,  Mesencephalon,  Cerebellum,  Spinal cord | Not lesioned | Not applicable | -  -  -  - | 9.1  10.6  7.1  6.3 | 0  0  0  0 | -  -  -  - | -  -  -  - | 2  3  3  0 | 0  0  0  0 |
| 37 | Adult | Germany | 2015 | Cerebral cortex,  Mesencephalon,  Cerebellum,  Spinal cord | Not lesioned | Not applicable | -  -  -  - | 3.9  6.2  1.9  4.9 | 0  0  0  0 | -  -  -  - | -  -  -  - | 4  3.5  5.2  4 | 0  0  0  0 |
| 38 | Adult | Germany | 2015 | Cerebral cortex,  Mesencephalon,  Cerebellum,  Spinal cord | Not lesioned | Not applicable | -  -  -  - | 6.5  8  5  5 | 0  0  0  0 | -  -  -  - | -  -  -  - | 7  7  6  4 | 0  0  0  0 |
| 39 | Adult | Germany | 2015 | Cerebral cortex,  Mesencephalon,  Cerebellum,  Spinal cord | Not lesioned | Not applicable | -  -  -  - | 10  7  12  1.4 | 0  0  0  0 | -  -  -  - | -  -  -  - | 6  6  6  5 | 0  0  0  0 |

Location: geographic location of samples in Brazil and non-altered controls from Germany. Year/season: year and/or season in which the samples were collected. C. callosum: corpus callosum. Trig. ganglion: trigeminal ganglion. *: not assessed. -: alteration not observed. +: alteration observed. N.a.: not available. Neuron.: neuronophagia.^a^ mean of immunostained cells in lesioned areas of the central nervous system of horses.
